# Acupuncture combined with western medicine for the treatment of hypertension: A protocol for an updated systematic review and meta-analysis

**DOI:** 10.1097/MD.0000000000026412

**Published:** 2021-06-25

**Authors:** Si-Han Wang, Jia-Tuo Xu, Xiao-Juan Hu, Ji Cui

**Affiliations:** a Basic Medical College; b Shanghai Innovation Center of TCM Health Service, Shanghai University of Traditional Chinese Medicine, Shanghai, China.

**Keywords:** acupuncture, electroacupuncture, hypertension, meta-analysis, systematic review

## Abstract

**Background:**

Hypertension is a kind of cardiovascular syndrome with the main clinical manifestation of continuous increase of systemic arterial blood pressure. Hypertension coexists with other cardiovascular risk factors and is an important risk factor for cardiovascular and cerebrovascular diseases. Acupuncture is an important part of Traditional Chinese Medicine intervention. The antihypertensive effect of acupuncture on hypertension is based on the neuroendocrine system, characterized by multichannel and multitarget. This study aims to provide latest and updated proof of systematic review to assess the effectiveness and safety of acupuncture for hypertension.

**Methods:**

We will systematically search 9 databases from their inceptions to February 2021. Only randomized controlled trials of acupuncture combined with western medicine in the treatment of hypertension will meet the inclusion criteria. The main outcome measures we focus on include clinical efficacy, syndrome efficacy, Traditional Chinese Medicine syndrome score, diastolic and systolic blood pressure changes, blood pressure variability, heart rate variability, pulse rate variability, and adverse reactions. The research screening, data extraction, and risk of bias assessment will be employed by 2 reviewers independently, and disagreement will be decided by a third senior reviewer. The Revman 5.3 software will be used for meta-analysis. The confidence of proof will be rated adopting grading of recommendations assessment, development and evaluation tool and methodological quality of this research will be assessed using assessment of multiple systematic reviews-2 and risk of bias in systematic reviews. The publication quality will be evaluated by preferred reporting items for systematic reviews and meta-analyses (PRISMA).

**Results:**

This systematic review (SR) will provide evidence-based medical evidence for hypertension therapy by acupuncture combined with western medicine and we will submit the findings of this SR for peer-review publication.

**Conclusions:**

This SR will provide latest and updated summary proof for assessing the effectiveness and safety of acupuncture for hypertension.

**Registration number:**

INPLASY 202150047

## 1 Introduction

Hypertension, one of the most common chronic diseases, is a kind of cardiovascular syndrome with the main clinical manifestation of continuous increase of systemic arterial blood pressure.^[1]^ Hypertension coexists with other cardiovascular risk factors, which is an important risk factor for cardiovascular and cerebrovascular diseases.^[2]^ It can damage the structure and function of important organs, such as heart, brain, and kidney, and eventually lead to functional failure of these organs.^[3]^ Hypertension is an important risk factor for cardiovascular disease, end-stage kidney disease, and all-cause death.^[4]^

In 2017, American Heart Association /American College of Cardiology guidelines redefined the diagnostic criteria of hypertension, which defined hypertension as ≥ 130 /80 mm Hg.^[5]^ In 2018, European Society of Cardiology /European Society of hypertension guidelines and Chinese hypertension guidelines continue to follow the previous standards, defining hypertension as ≥ 140 /90 mm Hg.^[6,7]^ Based on 140 /90 mm Hg, there were about 1.13 billion hypertensive patients in the world in 2015, and the prevalence of hypertension in adults was 30% to 45%.^[8]^ With the aging of the global population and the change of people's lifestyle, the prevalence of hypertension is still on the rise.^[9]^ It is estimated that there will be 1.5 billion hypertensive patients in the world by 2025.^[10]^ Studies have shown that the incidence rate of blood pressure in China has been increasing year by year, but the awareness rate, treatment rate, and control rate of hypertension patients are still at a low level.^[11]^ Hypertension has brought a heavy economic burden on the health of Chinese patients. A cohort study shows that about 750,000 cardiovascular deaths in Chinese people aged 35 to 79 are associated with uncontrolled hypertension.^[12]^

At present, the etiology of hypertension is considered to be closely related to genetic factors, environmental factors, vasoactive substances, sympathetic nervous system, renin angiotensin system, nitric oxide synthase/nitric oxide, endothelin, and insulin.^[13]^ At present, more and more evidences support that abnormal sensitivity and reactivity of peripheral blood vessels and structural changes of vascular wall are the main pathogenesis of hypertension.^[14]^ Modern studies have confirmed that hypertension is the result of abnormal vascular changes. In the early stage of hypertension, the whole body is characterized by small artery spasm. Long-term high blood pressure causes arteriosclerosis, subintimal hyaline degeneration, and proliferation or hypertrophy of middle smooth muscle cells, resulting in wall thickening and lumen stenosis, accompanied by the decline of vascular function, maintaining and developing high blood pressure, and further leading to ischemic injury of important target organs, such as heart, brain, and kidney.^[15]^

Studies have shown that every 20 mm Hg rise in systolic blood pressure or 10 mm Hg rise in diastolic blood pressure will significantly increase the risk of cardiovascular disease.^[16]^ The revised version of Chinese guidelines for the prevention and treatment of hypertension in 2018 clearly points out that the fundamental goal of hypertension is to reduce the total risk of cardiovascular, cerebrovascular, renal, and vascular complications as well as death.^[17]^ The target of reducing blood pressure should be lower than 140 /90 mm Hg in general patients, and can be further reduced to less than 130 /80 mm Hg in tolerable patients and some high-risk and above patients.^[18]^ Meta-analysis showed that for every 10 mm Hg decrease in systolic blood pressure or 5 mm Hg decrease in diastolic blood pressure, the risk of major adverse cardiovascular events was reduced by 20%, and the risk of all-cause death was reduced by 10% to 15%.^[19]^ Therefore, the control of blood pressure is particularly important.

In the specific treatment, cardiovascular risk assessment should be carried out first. Lifestyle intervention is the basic treatment for hypertension. On the basis of improving lifestyle, patients whose blood pressure still exceeds 140 /90 mm Hg and/or the target level should be given drug treatment.^[20]^ If the patient is diagnosed as high-risk and very high-risk, the drug treatment should be started immediately. At present, the main drugs for the treatment of hypertension include diuretics, calcium channel blockers, adrenergic receptor blockers, renin–angiotensin–aldosterone system inhibitors, and sympathetic nerve inhibitors. According to the overall risk and specific characteristics of patients and drugs, the corresponding drugs are selected for treatment.^[21]^

Modern medicine for the treatment of hypertension clearly points out that its fundamental goal is to reduce the total risk of cardiovascular, cerebrovascular, and renal complications and death, mainly through antihypertensive drugs to control blood pressure.^[22]^ However, in clinical practice, there are still some limitations and confusion, especially for the treatment of grade I hypertension, there is no clear evidence that drug treatment will bring obvious benefits. In addition, due to the unstable effect of antihypertensive drugs, side effects and drug resistance caused by long-term medication, the compliance of patients is greatly affected, and the control rate and standard rate of blood pressure are reduced.^[23]^

As an important part of Traditional Chinese Medicine (TCM) intervention, acupuncture therapy has the outstanding characteristics of noninvasive and non-gastrointestinal administration, and can be combined with internal treatment, but can make up for the deficiency of internal treatment.^[24]^ It is an significant part of TCM in hypertension therapy. A large number of clinical studies have proved that acupuncture has the effect of reducing blood pressure, and has the advantages of small side effects, low cost, simple and easy to be accepted by patients.^[25]^ Acupuncture can adjust Yin and Yang of human body, dredge Qi, and blood of meridians, regulate the function of viscera, relieve symptoms, and play the role of treatment and health care by stimulating acupoints on the body surface of meridians and viscera.^[26]^

Currently, a number of clinical trials have shown that acupuncture has good clinical efficacy and safety for the treatment of hypertension.^[27]^ Studies have shown that acupuncture can improve the function of cerebral cortex, enhance blood circulation in the brain, make blood vessels dilate, and blood flow unobstructed, and its mechanism is mainly related to the regulatory effect of acupuncture on nerves, immunity, and body fluids.^[28]^ In addition, acupuncture can relax nervous state, achieve the balance of excitement and inhibition, thus alleviate dizziness and headache symptoms, improve sleep, and reduce blood pressure. Some studies have confirmed that the antihypertensive effect of acupuncture may be related to the regulation of the rostral ventrolateral medulla area, the final central pathway regulating cardiovascular activity, the regulation of vascular endothelial related active factors, and the direct regulation of renin and angiotensin.^[28]^

Systematic review (SR) is a kind of literature synthesis method, aiming at a specific clinical problem, systematically and comprehensively collect the current published or unpublished studies, use the strict evaluation methods of evidence-based medicine and clinical epidemiology, screen out the literatures that meet the quality standards, and carry out qualitative or quantitative combined analysis to get the best comprehensive conclusion at present.^[29]^

Previous studies have reported that acupuncture combined with western medicine can be used to the treatment of hypertension.^[30]^ Currently, although several SRs have addressed this issue, however, its effectiveness is still inconclusive and none of them have further evaluated the effectiveness and safety of acupuncture for hypertension after more new randomized controlled trials (RCT) have been published.^[31]^ Therefore, this study aims to provide latest and updated evidence of systematic review to evaluate the effectiveness and safety of acupuncture for hypertension and provide reliable evidence for clinical practitioners.

## 2 Methods and analysis

### 2.1 Objective

This systematic review and meta-analysis aims to evaluate the effectiveness and safety of acupuncture combined with western medicine in the treatment of hypertension.

### 2.2 Study registration

This SR has been registered on International Platform of Registered Systematic Review and Meta-analysis Protocols (INPLASY, https://inplasy.com/?s=202150047) and will be reported according to Preferred Reporting Item for Systematic Review and Meta-analysis Protocols (PRISMA-P) guidance.

### 2.3 Inclusion and exclusion criteria

#### 2.3.1 Type of study

This SR will only include RCT of acupuncture combined with western medicine in the treatment of hypertension regardless of blind. The language of the included studies will be limited to Chinese and English, and at least one main outcome should be evaluated. The treatment group and the control group should be compared in the same period. Quasi-RCTs, secondary hypertension studies, literature review, case reports, duplicated publications, expert treatment experience summaries, animal experiments, cell experiments, and trials selected cases with other serious diseases and complications will be excluded.

#### 2.3.2 Type of participants

The participants should be diagnosed with primary hypertension by using clearly defined or internationally recognized criteria. The patient's age should be more than 18 years old without other serious diseases and complications. There are no restrictions on race or gender. Patients with secondary hypertension will be excluded.

#### 2.3.3 Type of interventions

Acupuncture or electroacupuncture combined with western medicine should be treated in treatment group and western medicine in control group. The treatment group and the control group should be treated with the same western medicine. Western medicine refers to drugs recommended by national guidelines or expert consensus, such as diuretics, calcium channel blockers, adrenergic receptor blockers, renin–angiotensin–aldosterone system inhibitors, sympathetic nerve inhibitors, etc.

#### 2.3.4 Type of outcome measurements

The main outcome measures included clinical efficacy, syndrome efficacy, TCM syndrome score, changes of diastolic and systolic blood pressure, changes of blood pressure variability, heart rate variability, pulse rate variability, and adverse reactions.

### 2.4 Search strategy

We will search systematically in the following 9 databases: PubMed, EMBASE (include MEDLINE),Cochrane Central Register of Controlled Trial, ovid, Web of Science, China National Knowledge Infrastructure, WangFang Database, Chinese Science and Technology Periodical Database, SinoMed. The language is limited to Chinese and English. In addition, we will also supplement the search for the following clinical trial registration platforms to confirm ongoing and completed RCTs: Clinical Trials.gov trials registry, Chinese Clinical Trial Registry, Current Controlled Trials, WHO clinical trials registry, The Australian New Zealand Clinical Trials Registry and Centre Watch. The retrieval time range is from their inceptions of each database to March 2021. Grey literature will also be searched to avoid omission. We will also manually search the references of published SR and related professional journals. In addition, we will consult experts in relevant fields to determine the completed but unpublished literature. The search strategy in PubMed can be found in Supplemental Digital Content () and it will be adjusted according to the characteristics of each database.

### 2.5 Studies selection and data extraction

**Figure 1:**
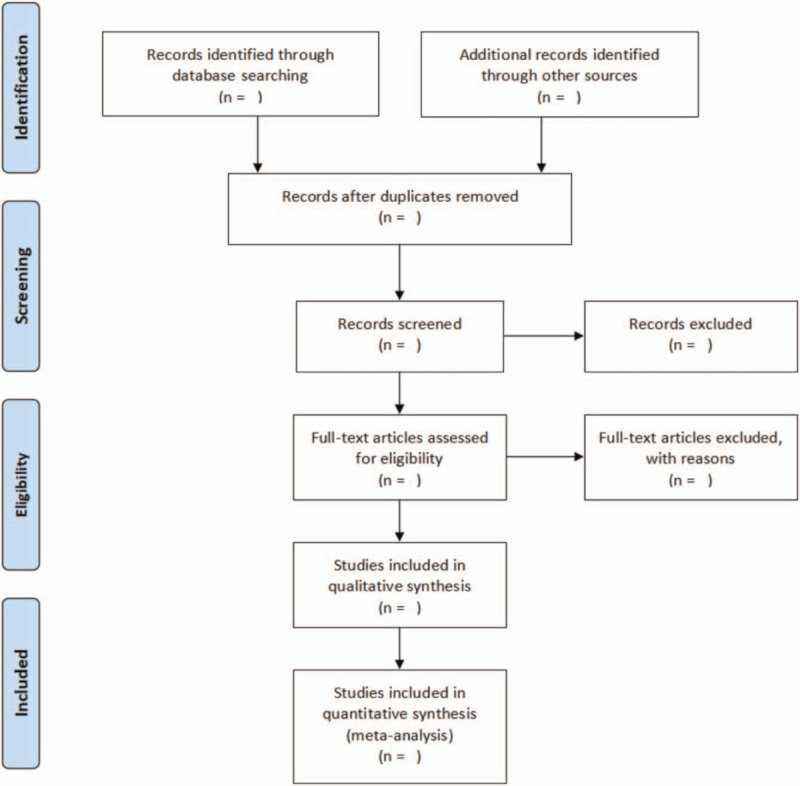
Flow diagram of study selection process. Details of the selection process will be presented in the PRISMA flow chart. PRISMA = preferred reporting items for systematic reviews and meta-analyses.

We will use EndNoteX 8.0 software to manage the retrieved literature. First, the retrieved literatures will be selected by 2 researchers independently according to the inclusion and exclusion criteria, and the full text of the literatures meeting the criteria will be downloaded for further screening. The researchers will record the literature excluded in this process and provide reasonable reasons. Details of the selection process will be presented in the PRISMA flow chart (Fig. 1).

Two researchers will extract information from the included eligible studies using a predetermined form. The key information extracted includes funding, participants (age, sex, course of disease), interventions (acupoints selection, frequency, duration), controls (type, measures, frequency, and duration), outcomes (frequency and duration, evaluation time points, and outcome measures) and study design (randomisation, allocation concealment, blinding, and etc). If required information is not reported, we will try to request it from the corresponding author of the studies.

Divergence in the process of literature selection and information extraction will be solved through discussion or judged by a third senior researcher. If the included RCTs have missing data, we will contact the corresponding authors. If missing data are unobtainable, intention-to-treat analysis will be conducted and sensitivity analysis will be performed to address the potential impact of missing data, which will be discussed if necessary.

### 2.6 Risk of bias and methodological quality assessment

Cochrane risk of bias assessment tool version 2.0 (RoB 2.0) will be used to assess the risk of bias of RCTs. We will evaluate the risk of bias in RCT from 5 domains, namely “risk of bias arising from the randomization process,” “risk of bias due to deviations from the intended interventions,” “missing outcome data,” “risk of bias in measurement of the outcome,” “risk of bias in selection of the reported result.”^[32]^

Each domain contains several signal questions and the evaluator may answer “probably yes,” “probably no,” “no,” “no information” according to the content of clinical study. According to the evaluation results of 5 areas, the overall risk of bias may be “low risk,” “possible risk,” and “high risk.”^[33]^

In addition, we will use the modified Jadad scale to evaluate the quality of the included studies. We will evaluate 4 aspects, namely “randomization,” “concealment of allocation,” “double blinding,” “withdrawals and dropouts.” According to the scoring principle, 1 to 3 scores will be considered as low-quality literature, and 4 to 7 scores as high-quality literature.

This process will be evaluated independently by 2 researchers, during which disagreement will be solved through discussion or judged by a third senior researcher.

### 2.7 Strategy for data synthesis

The Revman 5.3 software will be used for perform meta-analysis. The Q test and *I*^*2*^ values will be applied to measure the inter-study heterogeneity. When the *P* value of Q test >.1 and *I*^*2*^<50%, heterogeneity is acceptable and a fixed effects model will be applied. If *P* value of Q test <.1 and *I*^*2*^>50%, heterogeneity is significant and we will use subgroup analysis, meta regression analysis, and sensitivity analysis to explore the causes, if still unable to find, we will use random effect model to estimate or descriptive analysis. Binary variables will be expressed using the risk ratio with 95% confidence interval and continuous variables by the weighted mean difference with 95% confidence interval. We will use the method of eliminating low-quality research according to the risk of bias assessment results and replacement effect model to conduct sensitivity analysis to judge the robustness of the conclusion. If the number of included studies is more than 10, we will use inverted funnel plot to detect potential publication bias. Besides, Peters regression test will be applied for binary variables, and Egger for continuous variables to provide quantitative evidence of any publication bias.

### 2.8 Rating the confidence in estimates of the effect

We will use Grading of Recommendations Assessment, Development and Evaluation (GRADE) system to evaluate the confidence of outcome estimates.^[34]^ Five factors may downgrade the level of evidence, namely “inconsistency,” “imprecision,” “limitations/risk of bias,” “publication bias,” and “indirectness.” The overall quality of evidence may be rated as “high,” “moderate,” “low,” or “very low.” This process will be performed independently by 2 reviewers, during which disagreement will be solved through discussion or judged by a third senior reviewer.

### 2.9 Assessment of methodological quality and reporting quality

Assessment of multiple systematic reviews-2 (AMSTAR-2)^[35]^ and Risk of bias in systematic reviews (ROBIS^)^^[36]^ will be used to evaluate the methodological quality of this study. Furthermore, we will use PRISMA ^[37]^ to assess the reporting quality of this research. We will calculate kappa statistics to understand the consistency of assessments by AMSTAR-2, ROBIS, and PRISMA between 2 reviewers. Kappa 0.6 to 0.8 will be regarded as “substantial agreement” and 0.8 to 1.0 as “almost perfect agreement.” This process will be conducted independently by 2 researchers, and disagreement will be decided through discussion or by a third senior researcher.

### 2.10 Reporting standards

This systematic review and meta-analysis will be reported according to Reporting Items for Systematic Reviews and Meta-analyses of Acupuncture: the PRISMA for Acupuncture Checklist.^[38]^

This systematic review will not require ethical approval because there are no data used in our study that are linked to individual patient data. In addition, findings will be disseminated through peer-review publications.

### 2.11 Strengths and limitations of this study

1. This research will be the latest and updated SR to summarize the relevant proof of acupuncture combined with western medicine for the treatment of hypertension.
2. GRADE system will be applied to rate the confidence of evidence in this proof.
3. AMSTAR-2, ROBIS, and PRISMA will be used to evaluate the methodological quality and reporting quality of this research.
4. The search language is limited to Chinese and English, which may lead to omission.
5. This study will use a variety of ways to conduct a comprehensive search of potential RCTs.
6. This study will evaluate the results of different methods for the treatment of hypertension through autonomic nervous function evaluation indicators.
7. Due to the limitation of team members, we will only search Chinese and English electronic database.

## 3 Discussion

Human blood pressure regulation is mainly divided into short-term regulation and long-term regulation. Short-term regulation refers to the immediate regulation of blood pressure changes in a short period of time, mainly through neural regulation. The long-term regulation is mainly accomplished by the renal-body fluid control system, in which the renin–angiotensin system is the most important factor. The central nervous system regulates blood pressure by regulating sympathetic and parasympathetic nerves and their related neurotransmitters.^[39]^ The antihypertensive effect of acupuncture on hypertension is based on the neuroendocrine system, characterized by multichannel and multitarget.^[40]^ The comprehensive effect of acupuncture overcomes the shortcomings of single way of action and narrow indications of western medicine.^[41]^ We should further study and explore the antihypertensive effect of acupuncture, and establish a scientific evidence system of acupuncture in the treatment of hypertension, so as to provide a basis for the comprehensive treatment of hypertension in clinic.

## Author contributions

WS and XJ conceived and designed this protocol. WS drafted this article. HXJ and CJ reviewed and edited this article.

**Conceptualization:** Sihan Wang, Xu Jiatuo.

**Formal analysis:** Sihan Wang, Xu Jiatuo.

**Funding acquisition:** Ji Cui, Xiao-Juan Hu.

**Investigation:** Sihan Wang.

**Methodology:** Sihan Wang, Xu Jiatuo, Ji Cui.

**Project administration:** Sihan Wang, Xu Jiatuo, Ji Cui, Xiao-Juan Hu.

**Supervision:** Xiao-Juan Hu.

**Writing – original draft:** Sihan Wang.

**Writing – review & editing:** Sihan Wang, Xu Jiatuo, Ji Cui, Xiao-Juan Hu.

## Supplementary Material

Supplemental Digital Content
