# Supplementary material for: Acupuncture combined with western medicine for the treatment of hypertension: A protocol for an updated systematic review and meta-analysis
Source: Medicine (Baltimore). 2021 Jun 25;100(25):e26412. doi: 10.1097/MD.0000000000026412 (PMC8238323; doi:10.1097/MD.0000000000026412)
Supplement: Supplemental Digital Content [file medi-100-e26412-s001.docx]

| **Search Strategy in Pubmed:**  **#1** acupuncture[MeSH Terms] OR electroacupuncture[MeSH Terms] OR acupuncture with electric stimulation[MeSH Terms]  **#2** hypertension[MeSH Terms] OR high blood pressure[Title]  **#3** randomized controlled trial[Publication Type] OR randomized[Title/Abstract]) OR placebo[Title/Abstract] OR randomly[Title/Abstract] OR drug therapy [MeSH Subheading] OR trial [Title/Abstract] OR groups[Title/Abstract] OR meta[Publication Type] OR systematic review[Publication Type]  **#4** # 1AND #2 AND #3 |
| --- |
